# Chilaiditi Syndrome: A Case Report Highlighting the Intermittent Nature of the Disease

**DOI:** 10.1155/2018/3515370

**Published:** 2018-06-21

**Authors:** Esha M. Kapania, Christina Link, Joshua M. Eberhardt

**Affiliations:** Loyola University Medical Center, Maywood, IL, USA

## Abstract

**Background:**

Chilaiditi syndrome is a phenomenon where there is an interposition of the colon between the liver and the abdominal wall leading to clinical symptoms. This is distinct from Chilaiditi sign for which there is radiographic evidence of the interposition, but is asymptomatic.

**Case Presentation:**

Here, we present the case of a patient who, despite having clinical symptoms for a decade, had a delayed diagnosis presumably due to the interposition being intermittent and episodic.

**Conclusions:**

This case highlights the fact that Chilaiditi syndrome may be intermittent and episodic in nature. This raises an interesting question of whether previous case reports, which describe complete resolution of the syndrome after nonsurgical intervention, are perhaps just capturing periods of resolution that may have occurred spontaneously. Because the syndrome may be intermittent with spontaneous resolution and then recurrence, patients should have episodic follow-up after nonsurgical intervention.

## 1. Background

Chilaiditi syndrome is a rare condition in which there is radiographic evidence of colonic interposition between the liver and the diaphragm or abdominal wall, resulting in clinical symptoms [1]. The hepatic flexure of the colon is the portion of the bowel most commonly interposed; however, the small bowel has been implicated in a small number of cases [2]. The incidence is thought to be somewhere between 0.025 and 0.28% [1, 3, 4]. It is most common in males and presents at a median age of 60 [2, 5]. Due to its low prevalence, there is very little known about the etiology. It is thought that it can be congenital or acquired, with the former including a congenital absence of the falciform or suspensory ligaments, and the latter including factors such as cirrhosis, paralysis of the right diaphragm, and obesity [1]. There is also one case report illustrating the development of Chilaiditi syndrome after a colonoscopy [5].

While it presents with distinct radiographic findings, the rarity of the disease, along with variations in clinical presentation, often results in misdiagnosis or delayed diagnosis. In this case, we present a 73-year-old female who, despite having clinical symptoms for a decade, remained undiagnosed until recently. This case raises an interesting point that the syndrome may be intermittent in nature.

## 2. Case Presentation

**Figure 1:**
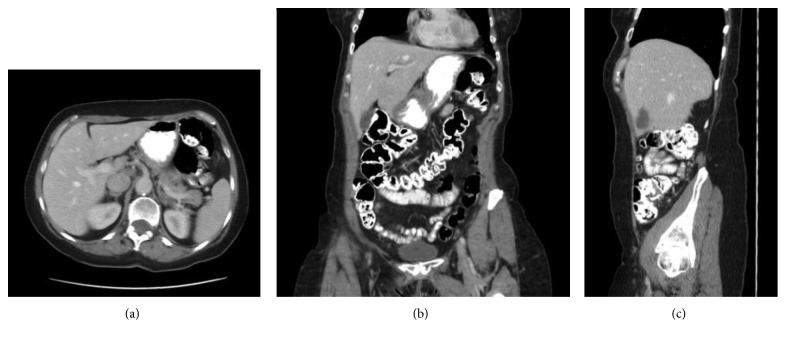
(a) Axial, (b) coronal, and (c) sagittal images from the patient's 2015 CT abdomen and pelvis showing no colon anterior to the liver.

**Figure 2:**
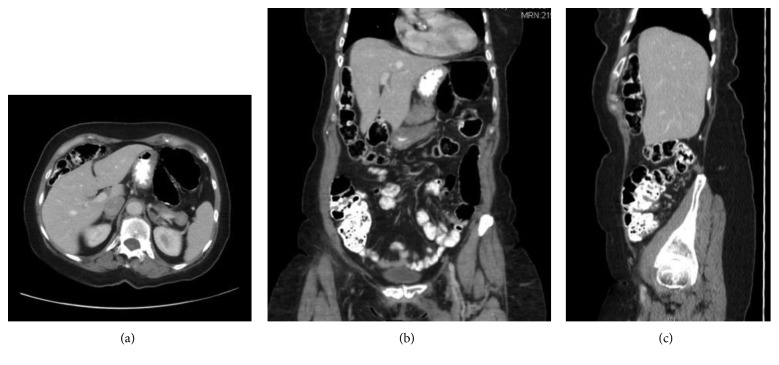
(a) Axial, (b) coronal, and (c) sagittal images from the patient's 2017 CT abdomen and pelvis demonstrating colon anterior to the liver.

A 73-year-old female with a past medical history of atrial fibrillation and mitral valve prolapse was referred due to intermittent right-sided abdominal pain and a right-sided abdominal bulge. Approximately 10 years ago, she experienced the initial onset of the symptoms which resolved spontaneously. This episode was temporally related to a severe coughing episode. Since then, the same pain and right-sided bulge would recur and then remit spontaneously. She went to an ED for these symptoms in both 2013 and 2015, and in both times, the evaluations, including a CT abdomen, were negative (Figure 1). A colonoscopy in 2016 was normal. In 2017, the symptoms recurred and prompted yet another ED visit. A CT at this time showed colon interposed between the liver and the abdominal wall along with some mild periappendiceal stranding (Figure 2).

Because of the long duration of symptoms which appeared to be increasing in frequency and severity and the radiologic findings, it was decided that the best course of action was surgical intervention. She underwent an uneventful laparoscopic right colectomy. During initial laparoscopic exploration, it was noted that the colon was no longer interposed above the liver, and the only abnormal gross finding was a very redundant proximal transverse colon which could easily be maneuvered into the configuration noted on her CT scan. The appendix was grossly normal on laparoscopic evaluation. The patient was discharged on postoperative day 3 with no complications. Incidentally, the final pathology showed a small invasive appendiceal adenocarcinoma arising in the background of goblet cell carcinoid with negative lymph nodes and negative margins staged as T3N0.

## 3. Discussion and Conclusion

Chilaiditi syndrome is rare and refers to an interposition of the colon between the liver and the abdominal wall resulting in clinical symptoms. The syndrome was named after Demetrious Chilaiditi, a Greek radiologist who first described three cases of the radiographic anomaly in 1911. Conversely, radiographic findings without the presence of any clinical symptoms are referred to as Chilaiditi sign [3]. The prevalence of the disease is thought to be somewhere between 0.025 and 0.28% [1, 3, 4].

Presentation can vary significantly. Many patients are completely asymptomatic and are diagnosed incidentally. In symptomatic patients, the most common presentation is abdominal pain, bloating, nausea, vomiting, and constipation [6]. In more severe cases, symptoms can extend to include difficulty breathing and chest pain, depending on the extent of colon involved [1]. While the disease can manifest itself with mild GI symptoms for decades without any complications, feared complications include the development of volvulus or perforations.

Diagnosis of the disease is made primarily through imaging, with CT scans being the imaging modality of choice [1, 3, 4]. The characteristic radiographic finding is the air below the diaphragm associated with visible haustra, which does not change with variation in patient position. Additional radiographic findings include elevation of the right hemidiaphragm above the liver by the intestine and depression of the superior margin of the liver below the left hemidiaphragm [1, 3, 4]. While Chilaiditi syndrome presents with very distinct radiographic findings, the rarity of the disease often results in misdiagnoses including diaphragmatic hernia, pneumoperitoneum, subphrenic abscesses, or inflammatory conditions.

Treatment for Chilaiditi syndrome varies depending on the severity of the symptoms. Patients who are asymptomatic do not require any intervention. Patients who present with mild or intermittent symptoms can often be treated initially with conservative management including bowel rest, IV fluids, bowel decompression, enemas, and laxatives [1, 3, 4]. In many patients who undergo this treatment, repeat CT scans show resolution of both the interposition and the clinical symptoms [1, 3, 4]. Surgical treatment is reserved for patients whose symptoms do not resolve with conservative management or for suspicion of a complication such as ischemia or perforation [2]. Surgical options range from pexy to colonic resection depending on the location of the interposition and the extent of bowel involved. For example, if the patient has evidence of involvement of the transverse colon, gangrene, or perforation, resection is recommended over pexy [2].

This case highlights the fact that the colonic interposition between the abdominal wall and liver may be episodic and intermittent in nature. This unfortunately adds an additional layer of complexity when it comes to diagnosing the problem and determining the best course of treatment. Furthermore, the potentially fleeting nature of the problem raises an interesting question: in previous reports where there is resolution of the syndrome after treatment, was the resolution attributable to the intervention or did it just resolve spontaneously? Perhaps, it is the natural history of the problem, at least in some cases, to come and go. Most reports, including ours, are limited by their lack of long-term follow-up. Finally, we believe that the appendiceal neoplasm in this case is incidental; however, we report it here in case further reports suggest an association in the future.
